# Diagnosis of CO_2_ dynamics and fluxes in global coastal oceans

**DOI:** 10.1093/nsr/nwz105

**Published:** 2019-08-02

**Authors:** Zhimian Cao, Wei Yang, Yangyang Zhao, Xianghui Guo, Zhiqiang Yin, Chuanjun Du, Huade Zhao, Minhan Dai

**Affiliations:** State Key Laboratory of Marine Environmental Science and College of Ocean and Earth Sciences, Xiamen University, Xiamen 361102, China

**Keywords:** CO_2_ dynamics and fluxes, coastal ocean, ocean-dominated margin, river-dominated ocean margin, carbon cycle

## Abstract

Global coastal oceans as a whole represent an important carbon sink but, due to high spatial–temporal variability, a mechanistic conceptualization of the coastal carbon cycle is still under development, hindering the modelling and inclusion of coastal carbon in Earth System Models. Although temperature is considered an important control of sea surface *p*CO_2_, we show that the latitudinal distribution of global coastal surface *p*CO_2_ does not match that of temperature, and its inter-seasonal changes are substantially regulated by non-thermal factors such as water mass mixing and net primary production. These processes operate in both ocean-dominated and river-dominated margins, with carbon and nutrients sourced from the open ocean and land, respectively. These can be conceptualized by a semi-analytical framework that assesses the consumption of dissolved inorganic carbon relative to nutrients, to determine how a coastal system is a CO_2_ source or sink. The framework also finds utility in accounting for additional nutrients in organic forms and testing hypotheses such as using Redfield stoichiometry, and is therefore an essential step toward comprehensively understanding and modelling the role of the coastal ocean in the global carbon cycle.

## INTRODUCTION

The coastal ocean, connecting the open ocean and the continents, provides important ecosystem services, particularly as a natural sink of atmospheric CO_2_ that contributes to ∼20% of the global ocean carbon uptake [1,2]. The coastal ocean is dynamic due to various physical and biogeochemical processes and its fluxes of carbon and other biogenic elements are highly variable in both time and space [3]. Quantifying the inventories and fluxes of coastal ocean carbon, essential for understanding the role of the global carbon cycle in climate variability and change, however, has been a long-standing challenge. As a result, the coastal ocean has been poorly represented in Earth System Models, in terms of both process understanding and temporal–spatial resolution [4,5]. Recent studies have further highlighted the susceptibility of coastal systems to increasingly intensified anthropogenic perturbations [6,7], adding to the challenge of understanding the already complex coastal carbon cycle and projecting its changes into the future.

Although available research has focused on carbon fluxes across several boundaries at the land–ocean interface, such as estuary–marsh interactions [3,8,9], knowledge of exchanges between ocean margins and the open ocean remains insufficient [3]. Carbon export from the coastal to open ocean was estimated as 0.75 Pg C yr^−1^ based on mass balance calculations, but a direct global estimate of the boundary exchanges between the coastal and open ocean is still unattainable due to data paucity [6]. The long-term wintertime data of the partial pressure of CO_2_ (*p*CO_2_) suggest a tendency of enhanced uptake of atmospheric CO_2_ in many shelf regions [10], which likely exports more dissolved inorganic carbon (DIC) to the open ocean. If this is a global trend, the uptake and export processes in coastal oceans should be included in future models of sea–air CO_2_ exchange [7]. In short, more efforts have focused on the river-estuarine input than the fundamental two-way or 3D exchange between ocean margins and the open ocean.

Furthermore, although the CO_2_ sink of coastal oceans is approaching a consensus value of 0.15–0.40 Pg C yr^−1^, whether a coastal ocean is a source or a sink of atmospheric CO_2_ varies vastly from one coastal system to another [11–19]. Based on a general latitudinal distribution featuring lower seawater *p*CO_2_ and thus strong sinks at high latitudes, and high seawater *p*CO_2_ and thus sources at low latitudes [12,17,20], temperature-induced changes in the solubility of CO_2_ have been considered a major control (i.e. thermal control) of coastal ocean CO_2_ fluxes. However, there are also other non-thermal factors at play, particularly water mass mixing (e.g. CO_2_ supplied by upwelling) and net primary production, resulting in deviations from this latitudinal distribution [17,19,21]. Understanding these processes in individual systems is a prerequisite for properly modelling the role of the coastal ocean in the global carbon cycle.

Thus, despite great efforts devoted to observing individual coastal systems [22–31] and synthesizing the global coastal CO_2_ fluxes [11–19] over the past decades, precisely modelling coastal ocean carbon either using regional models [32] or in a more comprehensive way integrating all domains of Earth’s surface [33] remains difficult, mainly due to unknown or oversimplified processes and fluxes in a highly dynamic setting [4,34]. Before an ultimate comprehensive model for predicting future coastal carbon trends at both regional and global scales can be developed, it is useful to establish a conceptual model of the coastal carbon cycle that makes full use of the limited available observations to advance our understanding of physical and biogeochemical processes.

In this study, we show the importance of non-thermal factors on coastal *p*CO_2_ and CO_2_ fluxes. We establish a semi-analytical framework to understand these non-thermal processes and to diagnose the CO_2_ source/sink nature of coastal oceans. By applying the framework to different coastal systems, we highlight the complexity of issues associated with coastal CO_2_ dynamics and fluxes, and demonstrate the utility of the framework, particularly in identifying pathways that advance our comprehensive understanding and modelling of the coastal carbon cycle.

## RESULTS AND DISCUSSION

### Non-thermal controls on coastal *p*CO_2_

**Figure 1.**
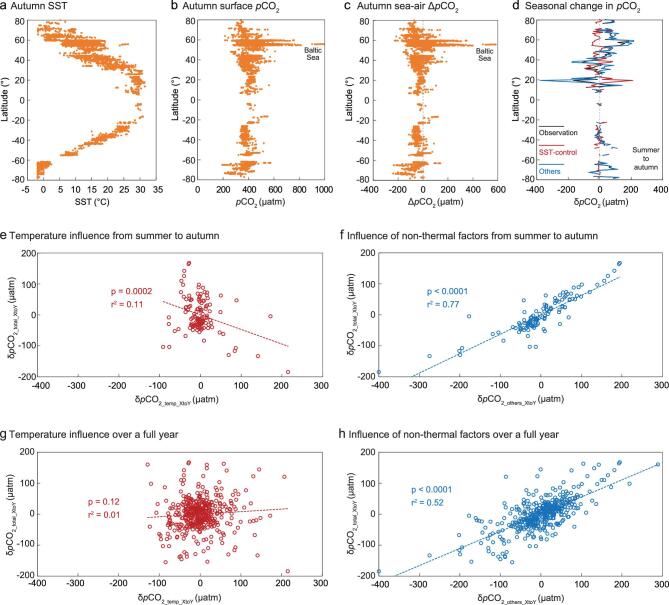
Evidence for non-thermal controls on partial pressure of CO_2_ (*p*CO_2_) in global coastal oceans. (a)–(c) Latitudinal distribution of sea surface temperature (SST), surface *p*CO_2_ and sea–air Δ*p*CO_2_ (defined as the difference in *p*CO_2_ between the sea and the air, or *p*CO_2_sea_ − *p*CO_2_air_) in autumn. (d) Seasonal change in *p*CO_2_ (δ*p*CO_2_) from summer to autumn, averaged over a 1°-latitude band; black, red and blue lines indicate δ*p*CO_2_ from field observations (δ*p*CO_2_total_XtoY_; XtoY means from X season to Y season; Equation (1)), solely due to temperature variations (δ*p*CO_2_temp_XtoY_; Equations (2) and (3)) and controlled by other factors (δ*p*CO_2_others_XtoY_; Equation (4)) such as mixing and biogeochemical processes. (e) and (f) Relationship of δ*p*CO_2_total_XtoY_ (black curve in (d)) to δ*p*CO_2_temp_XtoY_ (red curve in (d)) and δ*p*CO_2_others_XtoY_ (blue curve in (d)) from summer to autumn, based on departures from the average, showing that cooler SSTs in fact lead to a higher δ*p*CO_2_total_XtoY_, opposite to the argument of a temperature control over this seasonal transition. Instead, non-thermal factors, such as mixing and biogeochemical processes, control δ*p*CO_2_total_XtoY_. (g) and (h) Relationship of δ*p*CO_2_total_XtoY_ to δ*p*CO_2_temp_XtoY_ and δ*p*CO_2_others_XtoY_ between two consecutive seasons over a full inter-seasonal cycle, based on departures from their respective averages, highlighting an overall lack of control by SST changes but a strong influence of mixing and biogeochemical processes.

To illustrate the relative importance of temperature and non-thermal factors to coastal CO_2_ dynamics and fluxes, we use sea surface temperature (SST), *p*CO_2_ and sea–air Δ*p*CO_2_ (defined as the difference in *p*CO_2_ between the sea and the air, or *p*CO_2_sea_ − *p*CO_2_air_) (Fig. 1a–c) data collected at distances of 50 and 100 km from the global shoreline during all four seasons (Supplementary Figs 1, 2 and 3 and Supplementary Section 1.1; see the ‘Methods’ section). Similarly to previous studies synthesizing global coastal CO_2_ fluxes [10,17,19], these data mainly represent observations in continental margins consisting of shelf, slope and adjacent marginal seas and excluding nearshore ecosystems in internal waters such as estuaries, lagoons or tidal marshes. While SST clearly shows high values at low latitudes and low values at high latitudes, *p*CO_2_ and sea–air Δ*p*CO_2_ have a much less defined pattern during each season (Fig. 1a–c and Supplementary Figs 1 and 2). In autumn at 50 km from the shore, for instance, there is no clear latitudinal pattern of *p*CO_2_, with comparable average values between low and high latitudes (Fig. 1b). In the Baltic Sea at ∼55°N, *p*CO_2_ even approaches 1000 μatm. This leads to extremely high sea–air Δ*p*CO_2_ of ∼600 μatm, suggesting that this high-latitude coastal system is a CO_2_ source (Fig. 1b and c).

In addition to the latitudinal distribution of global coastal *p*CO_2_, which displays a mismatch to that of SST, we quantify the influence of temperature on *p*CO_2_ based on the experimental finding that ∂ln*p*CO_2_/∂T is 0.0423°C^−1^ for isochemical seawater [35,36]. Because it is nearly impossible to locate an isochemical seawater system, a reasonable approximation is to examine seasonal *p*CO_2_ changes in a system with predictable seasonal cycles. Comparing between the observed inter-seasonal *p*CO_2_ change (δ*p*CO_2_total_XtoY_; XtoY means from X season to Y season) and the temperature-controlled change (δ*p*CO_2_temp_XtoY_) gives the fractional amount of δ*p*CO_2_ that is not controlled by temperature (δ*p*CO_2_others_XtoY_; Equations (1)–(4); see the ‘Methods’ section). We infer that different latitudinal bands vary in their inter-seasonal controls (Fig. 1d and Supplementary Figs 1 and 2).

An examination of the seasonal changes from summer to autumn reveals that non-thermal factors prevail at all latitudinal bands, except from 25° to 35° in both hemispheres (Fig. 1d). This is supported by a statistically significant positive relationship between δ*p*CO_2_total_XtoY_ and δ*p*CO_2_others_XtoY_ (Fig. 1f). On the other hand, a statistically significant relationship between δ*p*CO_2_total_XtoY_ and δ*p*CO_2_temp_XtoY_ indicates that decreased SST is associated with enhanced δ*p*CO_2_total_XtoY_ (Fig. 1e), contrary to what is expected from the implied thermal control. This is reinforced by aggregating all δ*p*CO_2_ data over two consecutive seasons in a full inter-seasonal cycle (Fig. 1g and h). Compilations of the data at 100 km from the shore give a very similar pattern of *p*CO_2_ in terms of temperature and non-thermal determinations (Supplementary Fig. 2 and Supplementary Section 1.1). Consequently, the inter-seasonal change in global coastal *p*CO_2_ is to a large extent determined by non-thermal factors including water mass mixing and net primary production [17,19,21].

This analysis assumes that coastal systems are isochemical on a global scale; however, such systems are better approximated by more fully characterizing individual ocean margin systems. We thus differentiate the influence of temperature on *p*CO_2_ from the influence of non-thermal factors in the South China Sea and the Arabian Sea (see Supplementary Section 1.2).

In the South China Sea basin, temperature predominately controls the seasonal *p*CO_2_ change from winter to spring, but both temperature and non-thermal factors play important roles in the other three inter-seasonal changes. Taking the transition from spring to summer as an example, the temperature increase augments *p*CO_2_ by 17 ± 11 μatm, but is offset by the *p*CO_2_ decrease of −23 ± 17 μatm induced by non-thermal factors, leading to a negligible inter-seasonal *p*CO_2_ change of −6 ± 13 μatm (Supplementary Table 1). The latter change mainly reflects physical and biological processes including sea–air exchange, water mass mixing and biological activity.

In the Arabian Sea, the significant seasonal *p*CO_2_ increase from spring to summer in the coastal area mainly results from the summer upwelling process delivering CO_2_-rich deep water to the surface; temperature plays a minor role. Moreover, the subsequent decrease in upwelling largely induces the seasonal *p*CO_2_ decrease from summer to autumn, suggesting that water mass mixing is the major control. In areas without upwelling, both temperature and non-thermal factors play an important role in the inter-seasonal *p*CO_2_ changes (Supplementary Table 2).

Thus, non-thermal factors—mainly water mass mixing such as upwelling and biological alteration such as net primary production [17,19,21]—play a substantial role in regulating coastal ocean *p*CO_2_. The latitudinal distribution of *p*CO_2_ is obscured by seasonal changes in physical and biogeochemical processes at both river–margin and margin–open ocean interfaces, which cannot be fully simulated even by latest high-resolution models [10,32]; in contrast, such boundary processes are less important for open ocean basins. An alternative way to describe coastal CO_2_ dynamics is therefore required.

### Ocean-dominated margins versus river-dominated ocean margins

To depict the role of water mass mixing and net primary production, we conceptualize the coastal carbon cycle using a semi-analytical framework (see the ‘Methods’ section) that couples physics and biogeochemistry, and DIC and nutrients [16]. Such a semi-analytical framework highlights the boundary processes and aims to resolve the source of DIC and nutrients and their relative consumption via organic carbon production, which determines whether a coastal system is a source (i.e. an excess of DIC during consumption is removed by CO_2_ degassing into the atmosphere) or sink (i.e. a deficit of DIC during consumption is supplied via atmospheric CO_2_ input) of atmospheric CO_2_. In this context, the world’s coastal oceans are categorized into two distinct regimes: ocean-dominated margin (OceMar) and river-dominated ocean margin (RiOMar) [16,37], with DIC and nutrients sourced from the open ocean and land, respectively. OceMars are characterized by a concurrent non-local input of DIC and nutrients, typically from depth, and the interplay between the externally sourced DIC and nutrients through internal metabolism largely controls the CO_2_ source/sink nature. RiOMars are mostly shelf regions featuring major nutrient loadings from riverine inputs, which include the far-reaching area of river plumes of, for instance, the Amazon [38,39], Yangtze [27,40] and Mississippi [23,41], and exclude nearshore ecosystems such as estuaries, lagoons or tidal marshes. The large river plumes are characterized by high discharges and often show a drawdown of sea surface *p*CO_2_ resulting from increased net primary production. In these RiOMars, organic carbon production stimulated by riverine nutrients thus outweighs the remineralization of riverine organic matter, leading to sinks of atmospheric CO_2_.

Below, we verify our semi-analytical framework using new datasets compiled from recent observations in the South China Sea, previously identified as a typical OceMar system [16]. We then apply the framework to diagnose the CO_2_ source/sink nature of the Arabian Sea, which is a new OceMar case, and in the Pearl River plume—a major river plume system that is a RiOMar regime.

#### OceMar case I: South China Sea basin

The South China Sea is the largest marginal sea of the North Pacific Ocean and it is an overall source of atmospheric CO_2_ [25]. Our semi-analytical framework has successfully predicted the CO_2_ outgassing in its basin area, consistent with field observations during summer 2009 and spring 2011 [16]. The diapycnal fluxes of materials to the euphotic zone suggest a higher DIC flux relative to phosphate (PO_4_) than Redfield stoichiometry [42], further implying DIC excess in the South China Sea [43]. Here, we reassess the CO_2_ source/sink nature using new data collected in autumn (November) 2010 and summer (May–July) 2014 in the South China Sea basin (see Supplementary Section 1.3).

**Figure 2.**
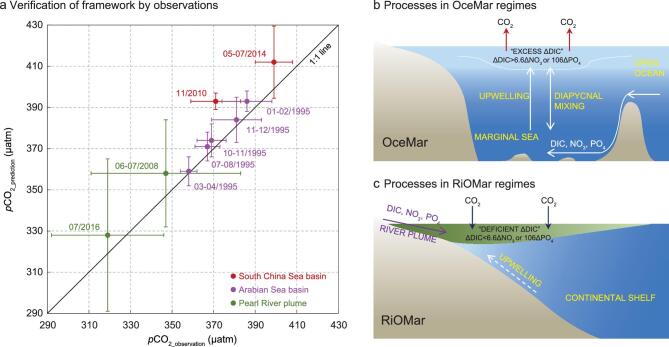
Application of our semi-analytical framework to ocean-dominated margins (OceMars) and a river-dominated ocean margin (RiOMar). (a) Verification of predicted *p*CO_2_ using our semi-analytical framework (see the ‘Methods’ section) versus the observed *p*CO_2_ in three applications: the South China Sea basin (red, two seasons), Arabian Sea basin (pink, five seasons) and Pearl River plume (green, two seasons). Horizontal and vertical bars represent one standard deviation of *p*CO_2_ values, reflecting the spatial variability of field observations and variability of our predictions, respectively. The South China Sea basin and the Arabian Sea basin, both diagnosed as a source of atmospheric CO_2_ by our framework, are OceMars. In these systems, DIC and nutrients are mainly from processes at the coastal and open ocean interface, whereby the open ocean-originated DIC, NO_3_ and PO_4_ are transported upward into the surface mixed layer of the marginal sea through vertical mixing and upwelling, as depicted by (b). By contrast, the Pearl River plume, diagnosed as a sink of atmospheric CO_2_ by our framework, is a RiOMar. In these systems, DIC, NO_3_ and PO_4_ mainly originating from rivers are transported along the plume pathway over the continental shelf, whereas contributions from the subsurface water play a minor role, as depicted by (c). The coupled DIC and nutrient consumption via organic carbon production in the surface mixed layer, or in the plume water, ultimately determine the sea–air CO_2_ flux of ocean margins. (b) and (c) are revised from [16].

Significant positive relationships between total alkalinity (TAlk) and salinity are observed in the surface mixed layer (<50 m) in both seasons, suggesting a two-endmember mixing scheme between waters immediately below the surface mixed layer and rain water, indicated by near-zero intercepts (Supplementary Fig. 7). Within this scheme, the estimated δDIC* based on PO_4_ (δDIC*_PO4_; Equation (11)) is on average 7 ± 2 μmol kg^−1^ in autumn 2010 and 17 ± 9 μmol kg^−1^ in summer 2014, which point to excess DIC removed by CO_2_ degassing, and are transformed to a sea–air Δ*p*CO_2_ of 13 ± 4 and 32 ± 18 μatm using Equation (14) (Supplementary Table 3). Combined with the atmospheric *p*CO_2_ field observations of ∼380 μatm in both seasons, the sea surface *p*CO_2_ (Equation (15)) in the South China Sea basin is predicted to be 393 ± 4 μatm in autumn 2010 and 412 ± 18 μatm in summer 2014, consistent with, if only slightly higher than, the field observations of 371 ± 12 and 399 ± 9 μatm (red-filled circles, Fig. 2a; Supplementary Fig. 8). Note that the estimated sea surface *p*CO_2_ based on nitrate (NO_3_) is essentially consistent with that based on PO_4_ in both seasons, which is discussed later. We thus confirm the CO_2_ source nature of the South China Sea.

The main processes involved in an OceMar regime are depicted in Fig. 2b. DIC and nutrients are mainly sourced from the open ocean. Through vertical mixing and upwelling, such open ocean-originated DIC, NO_3_ and PO_4_ are transported upward into the surface mixed layer, in which the consumption of DIC relative to nutrients determines whether DIC is in excess or in deficit relative to the external input. The excess DIC, as diagnosed in the South China Sea basin, is eventually released to the atmosphere, making the region a CO_2_ source.

#### OceMar case II: Arabian Sea basin

The Arabian Sea is characterized by strong seasonal cycles driven by the Asian Monsoon. There is well-defined upwelling during the southwest monsoon season due to offshore Ekman transport, which often manifests as low SST along the Arabian Peninsula [44]. The upwelling results in increased biological productivity and complicated *p*CO_2_ responses. Using the carbonate system and nutrient data collected in 1995 from five cruises in winter (January–February and November–December) during the northeast monsoon season, in spring (March–April) and autumn (October–November) during the inter-monsoon season and in summer (July–August) during the southwest monsoon season (see Supplementary Section 1.4), we estimate the CO_2_ source/sink nature in the Arabian Sea.

TAlk and salinity in the surface mixed layer (<75–100 m) show significant positive relationships in all seasons, suggesting an overall two-endmember mixing scheme between waters immediately below the surface mixed layer and rain water (indicated by near-zero intercepts) or a non-zero-solute freshwater (indicated by substantive intercepts) (Supplementary Fig. 10). Within this scheme, the average δDIC*_PO4_ values are estimated to be 23 ± 3 (January–February), 4 ± 5 (March–April), 20 ± 5 (July–August), 25 ± 6 (October–November) and 26 ± 8 (November–December) μmol kg^−1^, which point to excess DIC removed by CO_2_ degassing and are transformed to a sea–air Δ*p*CO_2_ of 37 ± 5, 6 ± 7, 30 ± 7, 37 ± 8 and 39 ± 11 μatm using Equation (14) (Supplementary Table 3). Sea surface *p*CO_2_ (Equation (15)) estimated for the five consecutive seasons are 393 ± 5, 359 ± 7, 371 ± 7, 374 ± 8 and 384 ± 11 μatm. The field-observed *p*CO_2_ values are 386 ± 12, 358 ± 4, 367 ± 6, 369 ± 7 and 381 ± 12 μatm, which agree rather well with the predicted results (pink-filled circles, Fig. 2a; Supplementary Fig. 11). Note that our analysis based on NO_3_ is, within error, comparable to that based on PO_4_ during the five seasons, which is discussed later. While the sea surface *p*CO_2_ equals the air *p*CO_2_ in the spring inter-monsoon season, in other seasons, the Arabian Sea must have acted as a weak source of CO_2_ to the atmosphere (Fig. 2b).

#### RiOMar case: Pearl River plume

The Pearl River is one of the world’s major rivers, discharging 3.26 × 10^11^ m^3^ of freshwater annually and forming a river plume over the broad continental shelf of the northern South China Sea in summer. This river plume, travelling along with the coastal current, extends to hundreds of kilometres from the mouth of the Pearl River estuary and strongly modulates the shelf biogeochemistry [45–47]. Here, we use datasets collected on the northern South China Sea shelf in the summers of 2008 (June–July) and 2016 (July) to evaluate the CO_2_ source/sink nature of the Pearl River plume waters with salinities <33.0 (see Supplementary Section 1.5).

Due to the co-influence of both the river plume and coastal upwelling in both seasons, a three-endmember mixing scheme in the upper 100 m is identified between the plume water, offshore subsurface water and offshore surface water, as suggested by the temperature–salinity relationship (Supplementary Fig. 13). Within this scheme, the estimated δDIC*, based on NO_3_ (δDIC*_NO3_; Equation (10)), is on average −6 ± 13 μmol kg^−1^ in summer 2008 and −34 ± 20 μmol kg^−1^ in summer 2016, which indicates a DIC deficit supplied via atmospheric CO_2_ inputs, and these values are transformed to a sea–air Δ*p*CO_2_ of −12 ± 26 and −62 ± 37 μatm using Equation (13) (Supplementary Table 3). Combined with the field-observed atmospheric *p*CO_2_ of ∼370 and ∼390 μatm during the two seasons, the sea surface *p*CO_2_ was estimated to be 358 ± 26 and 328 ± 37 μatm (Equation (15)); both values are consistent with the calculated *p*CO_2_ of 347 ± 36 and 319 ± 27 μatm using DIC and TAlk data (green-filled circle, Fig. 2a; Supplementary Fig. 14). We thus demonstrate that the Pearl River plume is a sink of atmospheric CO_2_, which was stronger in summer 2016 than in summer 2008. However, our analysis based on PO_4_ suggests it was a CO_2_ source during both seasons. This inconsistency is examined later.

The main processes involved in a RiOMar regime are depicted in Fig. 2c. DIC and nutrients are mainly sourced from the river and transported along the plume pathway on the continental shelf. As in OceMars, the consumption of DIC relative to nutrients in the plume water determines whether DIC is in excess or in deficit. A DIC deficit, as identified in the Pearl River plume, is supplied by atmospheric CO_2_ inputs, making the region a CO_2_ sink.

Thus, we successfully extend the applicability of the semi-analytical framework from OceMars to RiOMars, helping to better characterize the coastal carbon cycle and resolve the CO_2_ dynamics and fluxes in a quantitative way. While the major source of DIC and nutrients differs between the two coastal systems due to different boundary processes, the same metabolic process coupling the externally supplied DIC and nutrients modulates the sea–air CO_2_ exchange in both regimes (Fig. 2b and c).

**Figure 3.**
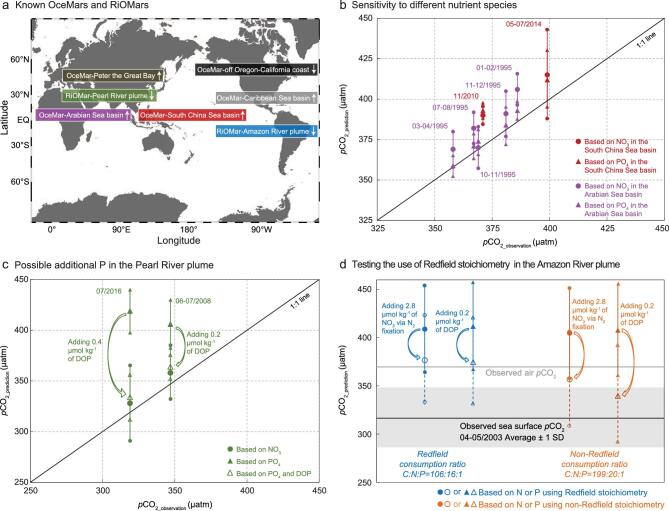
Utilities and uncertainties of our semi-analytical diagnostic framework in OceMar and RiOMar regimes. (a) Known OceMars and RiOMars, with upward and downward arrows indicating sources and sinks of atmospheric CO_2_, respectively. (b) Comparison between predicted *p*CO_2_ based on different nutrient species and observed *p*CO_2_ in the South China Sea basin (red, two seasons) and the Arabian Sea basin (pink, five seasons). The diagonal line indicates a perfect fit between the diagnosed and observed values, and vertical bars represent one standard deviation (1 SD) of the diagnosed *p*CO_2_ values, which are largely due to the spatial variability of the observations. The consistency between CO_2_ source or sink diagnoses based on NO_3_ or PO_4_ demonstrates insensitivity to the choice of nutrient species. (c) As in (b), but for the Pearl River plume (green, two seasons); in both seasons, our framework identifies an addition of P that is not accounted for by our observations but needs to be considered to bring the predicted *p*CO_2_ to that based on NO_3_. The added P is likely transformed from dissolved organic phosphorus (DOP). (d) As in (b), but for the Amazon River plume (blue, one season), which our framework diagnoses as a source, opposite to the observed sink. Under the Redfield stoichiometry, even with unaccounted-for N or P added, our framework still predicts a sea surface *p*CO_2_ (blue) higher than the observed air *p*CO_2_ (grey line) but, under a non-Redfield stoichiometry, the same added nutrients lead to a predicted *p*CO_2_ (orange) that is close to or within the uncertainty range due to spatial variability (grey shadow) of the observed average sea surface *p*CO_2_ (black line).

### Utilities and uncertainties

Our framework (see the ‘Methods’ section) characterizes two important carbon processes that are independent of temperature, namely physical and biogeochemical processes, in the coupled DIC–nutrient dynamics. Application of the framework strengthens the notion that, even though global coastal oceans are a sink of atmospheric CO_2_ as a whole, individual coastal systems are highly variable, may act as CO_2_ sources or sinks and need to be characterized individually. In addition to the three cases we presented here, other successful applications of our framework include the Caribbean Sea basin [16], the upwelling system of the US west coast off Oregon and California [48] and the Peter the Great Bay of the Japan/East Sea [30] (Fig. 3a). These are identified as OceMars and can be either a CO_2_ source or sink. However, for the Amazon River plume, a typical RiOMar system (Fig. 3a), our prediction as a source differs from observations as a sink. This is examined later.

The semi-analytical framework follows Redfield stoichiometry, in which the C, N and P consumption ratio is constant at 106:16:1 [42]. Treating this ratio as a global or regional constant is mostly acceptable

in the context of interpreting snapshots of the water column [49]. However, higher DIC/NO_3_ consumption ratios than Redfield stoichiometry have been observed, probably resulting from DIC overconsumption relative to inorganic N via the production of dissolved organic matter (DOM) [50]. This suggests that the assumption of a Redfield consumption ratio may not be always valid and must be verified. On the other hand, some of the unaccounted-for nutrients may also result in an apparently higher DIC/NO_3_, DIC/PO_4_ or NO_3_/PO_4_ consumption ratio [46].

Application of our framework to the South China Sea and the Arabian Sea illustrates that, when all nutrients are accounted for, our analysis is robust and insensitive to the choice of nutrient species. In the South China Sea basin, the estimated δDIC*_NO3_ agrees well with the δDIC*_PO4_ in both autumn 2010 and summer 2014, resulting from an average ΔNO_3_/ΔPO_4_ ratio (i.e. the apparent biological consumption of NO_3_ relative to PO_4_ obtained by Equation (8)/Equation (9)) close to the Redfield value of 16 (Supplementary Table 3). Accordingly, the estimated *p*CO_2_ values based on δDIC*_NO3_ and δDIC*_PO4_, respectively, are within error, comparable to each other during both seasons (red-filled symbols, Fig. 3b). In the Arabian Sea basin, the estimated *p*CO_2_ based on δDIC*_NO3_ is also within the uncertainty range of that based on δDIC*_PO4_, and both are consistent with field observations during the five seasons analysed (pink-filled symbols, Fig. 3b), when the ΔNO_3_/ΔPO_4_ ratios are equal to the Redfield value (Supplementary Table 3). The consistency between results from the two nutrient species suggests that consumption of C/N and C/P are well coupled in these two OceMar systems, which points to the robustness of our framework and strengthens the validity of using Redfield stoichiometry in these cases.

In the Pearl River plume, however, the estimated δDIC*_PO4_ values are positive in both the summers of 2008 and 2016 based on our own observations, which would imply a CO_2_ source (large green-filled triangles, Fig. 3c), opposite to the direction of the CO_2_ flux based on δDIC*_NO3_ (large green-filled circles, Fig. 3c). This discrepancy is supported by much higher ΔNO_3_/ΔPO_4_ ratios than the Redfield value (Supplementary Table 3), suggesting that an additional source of P not accounted for by our observations is needed to balance the NO_3_-based δDIC* and thus reduce the estimated *p*CO_2_ toward that based on δDIC*_NO3_ or field observations. This amount of P is estimated to be ∼0.2 and ∼0.4 μmol kg^−1^ for the summers of 2008 and 2016, respectively. Once added, the calculated *p*CO_2_ (large green-unfilled triangles, Fig. 3c) is close to the observed *p*CO_2_. The additional P can be transformed from dissolved organic phosphorus (DOP) during metabolic processes [46]. This finding of unaccounted-for DOP is confirmed by an independently observed value of approximately 0.5 μmol kg^−1^ in the upstream Pearl River estuary in summer 2015 [51], highlighting a utility of our framework.

Nevertheless, there is at least one case—the Amazon River plume—in which, even after accounting for additional nutrients, the predicted and observed results of sea surface *p*CO_2_ still differ. In this case, the debate regarding the validity of Redfield stoichiometry continues [50,52]. Here, using available observations of DIC, NO_3_ and PO_4_ (see Supplementary Section 1.6), our framework determines that the Amazon River plume (salinities ∼30.0–35.0) is a source of atmospheric CO_2_, whereas observations from April–May 2003 show it acts as a sink [38,39].

To be consistent, it would require a NO_3_ consumption of ∼7.4 μmol kg^−1^ to meet the DIC removal and drawdown of *p*CO_2._ The estimated ΔNO_3_ using our framework (Equation (8)) is, however, only ∼1.0 μmol kg^−1^ (the resulting prediction of *p*CO_2_ is denoted by a large blue-filled circle, Fig. 3d), suggesting that the majority of NO_3_ (∼6.4 μmol kg^−1^) needs to be supplied from additional sources, such as N_2_ fixation. Strong N_2_ fixation was consistently observed in the Amazon River plume [53–55], estimated to be 1404.6 μmol N m^−2^ d^−1^ in the mesohaline zone with salinities of 30.0–35.0 [56]. Combined with a mixed layer depth of ∼20 m and a water residence time of ∼40 days, the total N supplied by N_2_ fixation would only be 2.8 μmol kg^−1^, of which just a fraction could be directly transformed to NO_3_ and subsequently consumed during primary production. Even taking this value as a maximum of NO_3_ added, the resulting prediction of sea surface *p*CO_2_ is still higher than the field-observed atmospheric *p*CO_2_ (the former denoted by a large blue-unfilled circle, Fig. 3d). We therefore use another nutrient species, i.e. PO_4_, which leads to a finding that the estimated ΔPO_4_ (Equation (9)) is only ∼0.02 μmol kg^−1^ (the resulting prediction of *p*CO_2_ is denoted by a large blue-filled triangle, Fig. 3d) and additional PO_4_ of 0.53 μmol kg^−1^ is needed to predict the CO_2_ sink. DOP, with a concentration of up to 0.2 μmol kg^−1^, is the dominant species of P [57] (the resulting prediction of *p*CO_2_ is denoted by a large blue-unfilled triangle, Fig. 3d), which is still not sufficient to support the DIC consumption.

Although N_2_ fixation and DOP utilization may partially be responsible for the low NO_3_ and PO_4_ removal relative to DIC, including them in the semi-analytical framework seems incapable of reversing the prediction of their CO_2_ source/sink nature. We envisage that this could be a case in which Redfield stoichiometry is not fully valid. If DIC overconsumption occurred via the DOM production with higher C:N:P stoichiometry (199:20:1) [58], this would also lead to the overestimated δDIC*_NO3_ and δDIC*_PO4_ (Equations (10) and (11)). By considering both unaccounted-for nutrients and the non-Redfield stoichiometry of DOM, we can resolve the sea–air Δ*p*CO_2_ close to the CO_2_ sink nature of the Amazon River plume. In particular, the estimated *p*CO_2_ based on P is closer to the field-observed range of *p*CO_2_ (orange symbols, Fig. 3d). Nevertheless, this is obviously an oversimplification because DIC and inorganic nutrients are not merely transformed into DOM during biological consumption. The realistic C, N and P consumption ratio would lie in between, pointing to an uncertainty of our estimation, which warrants further study.

## SUMMARY

Although global coastal oceans as a whole have been recognized as an important natural sink of atmospheric CO_2_, the associated physical and biogeochemical processes need to be better understood. Our finding that inter-seasonal changes of global coastal *p*CO_2_ are strongly determined by non-thermal factors has led to the establishment of our semi-analytical framework, which conceptualizes the role of water mass mixing and net primary production. The framework diagnoses CO_2_ dynamics and fluxes in coastal oceans in two distinctive regimes, i.e. OceMars and RiOMars. We show that coastal oceans as a CO_2_ sink mostly refers to RiOMars, but OceMars can be either a source or sink: e.g. the South China Sea basin is a source whereas the Oregon–California coast is a sink. Therefore, the details are critical and must be captured correctly by any global carbon cycle models, which has been a long-standing scientific challenge.

It is in this context that our framework has found utility while contributing to the scientific debate. On the one hand, when validated by independent observations, it helps account for additional nutrients as is the case in the Pearl River plume. On the other hand, it helps test the validity of Redfield stoichiometry as is the case in the Amazon River plume. We anticipate that the usefulness of our developed framework will inspire future studies. One natural next step would be to apply this framework to other global coastal systems. To this end, observations of DIC and nutrients from likely sources are essential, but this is not a trivial effort. International collaboration is crucial in order to gain the global picture that is composed of detailed characteristics of individual coastal ocean systems. This would also increase understanding of the associated processes, and their parameterization for inclusion in models: either mechanistic, such as our framework, or as a comprehensive global system [33], the latter being the ultimate goal of future research efforts.

## METHODS

### Global coastal *p*CO_2_ data sources and analysis

Global coastal SST and *p*CO_2_ data employed in Fig. 1 and Supplementary Figs 1 and 2 were extracted from the Surface Ocean CO_2_ Atlas (http://www.socat.info/). We selected *p*CO_2_ values at the distance of 50 and 100 km from major land masses in the SOCAT v3.0 data product [59], which cover latitudes from 82°N to 78°S and years from 1962 to 2014 (Supplementary Fig. 3).

Data analysis was based on 1°-latitude averages. To differentiate the influence of temperature on seasonal *p*CO_2_ variations from all other influences, we first calculated the total variation of field-observed *p*CO_2_ from X season to Y season (δ*p*CO_2_*total*_*XtoY*_) according to Equation (1):

(1) \begin{equation*} \delta { {p\rm CO}}_{2\_{\mathit {total}}\_ XtoY}={{p\rm CO}}_{2\_Y}-{{p\rm CO}}_{2\_X}. \end{equation*}

*p*CO_2_*X*_ and *p*CO_2_*Y*_ are the 1°-latitude averaged field-observed *p*CO_2_ in X season and Y season, respectively. We then defined δ*p*CO_2_*temp_XtoY*_ as the *p*CO_2_ variation solely induced by temperature changes from X season to Y season, which was calculated as:

(2) \begin{equation*} \delta {{p\rm CO}}_{2\_ {\mathit {temp}}\_ XtoY}={{p\rm CO}}_{2\_\mathit {@ Y temp}}-{{p\rm CO}}_{2\_X}. \end{equation*}

*p*CO_2_*@Ytemp*_ is the theoretical *p*CO_2_ value during Y season considering only temperature changes relative to X season, which was calculated according to Equation (3) [36]:

(3) \begin{equation*} {p\rm CO}_{2\_\mathit {@ Ytemp}}={p\rm CO}_{2\_X}\nonumber\\ \times \exp \left[0.0423\times \left(\mathit{temp}_{\_Y}-\mathit{temp}_{\_X}\right)\right]. \end{equation*}

*temp__X_* and *temp__Y_* are the 1°-latitude averaged SSTs during seasons X and Y, respectively. Finally, the difference between δ*p*CO_2_*total_XtoY*_ and δ*p*CO_2_*temp_XtoY*_ (δ*p*CO_2_*others_XtoY*_; Equation (4)) reflects the *p*CO_2_ variability between seasons X and Y resulting from other processes (e.g. water mass mixing and net primary production) beyond the temperature effect.

(4) \begin{eqnarray*} \delta {p\rm CO}_{2\_\mathit {others\_ XtoY}}&=&\,\delta {p\rm CO}_{2\_ \mathit {total\_ XtoY}}\nonumber\\ &-&\delta {p\rm CO}_{2\_ \mathit {temp\_ XtoY}}. \end{eqnarray*}

The four seasonal transitions presented in Supplementary Figs 1 and 2 are changes sequentially from winter (X) to spring (Y), from spring (X) to summer (Y), from summer (X) to autumn (Y) and from autumn (X) to winter (Y). Seasons are defined as March, April and May as spring in the Northern Hemisphere and September, October and November as spring in the Southern Hemisphere.

### Semi-analytical diagnostic framework of CO_2_ dynamics and fluxes in ocean margins

Our diagnostic framework uses the mass balance of DIC in the surface mixed layer (Equation (5)):

(5) \begin{equation*} \frac{\mathit {\partial DIC}}{\partial t}=\sum {F}_\mathit {DIC}\!-\! \mathit {NEC}\!-\! \mathit {NEP}\!-\!{F}_{{\mathit CO}_2}.\end{equation*}

Here, *NEC* is the net ecosystem calcification and *NEP* is the net ecosystem production. *F_DIC_* is the external DIC input to the surface mixed layer and *F_CO_2__* is the sea–air CO_2_ flux. At steady state and assuming negligible net calcification often suggested by conservative TAlk distributions, Equation (5) can be simplified to Equation (6):

(6) \begin{equation*} {F}_{{\mathit CO}_2}=\sum {F}_\mathit {DIC}- \mathit {NEP}. \end{equation*}

The sea–air CO_2_ flux is thus a function of the sum of DIC inputs and net ecosystem production, or the CO_2_ is a net consequence of externally transported DIC and internal metabolic CO_2_ consumption. However, resolving Equation (6) is not a trivial task, as it requires a full solution to the 3D ocean circulation that is not always possible. Here, we couple the physical transport and biological mediation of DIC and nutrients (i.e. NO_3_ and PO_4_) within comparable timescales, by establishing a water mass mixing scheme in order to define the physical transport, or the conservative portion of DIC and nutrients from the adjacent water masses, and the constraint of the biogeochemical alteration of these non-local inputs in the upper water column of ocean margins [16]. These processes are described by

(7) \begin{equation*} \Delta \mathit {DIC}=\mathit {DIC}_\mathit {cons}-\mathit {DIC}_\mathit {meas}, \end{equation*}

(8) \begin{equation*} \Delta \mathit {NO}_3=\mathit {NO}_{3\_ \mathit {cons}}-\mathit {NO}_{3\_\mathit {meas}}, \end{equation*}

(9) \begin{equation*} \Delta \mathit {PO}_4=\mathit {PO}_{4\_ \mathit {cons}}-\mathit {PO}_{4\_ \mathit {meas}}. \end{equation*}

The subscripts ‘cons’ and ‘meas’ in Equations (7)–(9) denote conservative-mixing-induced and field-observed values, respectively. The difference between them, represented as Δ, is the addition (negative values resulting from organic matter remineralization outweighing primary production) or removal (positive values resulting from primary production outweighing organic matter remineralization) of DIC, NO_3_ or PO_4_ beyond the mixing control. In the latter case, we subsequently use δDIC^*^ to quantify the consumption of DIC relative to nutrients based on the classic Redfield stoichiometry of C:N:P = 106:16:1 (Equations (10) and (11)) [42], which determines whether DIC is in excess (i.e. δ*DIC*$^*$ >0) or in deficit (i.e. δ*DIC*$^*$ <0) relative to nutrients in the seawater body:

(10) \begin{equation*} \delta \mathit {DIC}^{\ast}=\Delta \mathit {DIC}-6.6\Delta \mathit {NO}_3, \end{equation*}

(11) \begin{equation*} \delta \mathit {DIC}{^\ast}=\Delta \mathit {DIC}-106\Delta \mathit {PO}_4. \end{equation*}

Under steady-state conditions over a relatively long timescale (e.g. seasonal scale), such excesses or deficits in DIC would be degassed (i.e. CO_2_ source) or compensated (i.e. CO_2_ sink) by sea–air CO_2_ gas exchange. At the same time, only a fraction of δDIC^*^ is involved in the gas exchange, depending on the Revelle factor (RF), which is referred to as the fractional change in surface seawater CO_2_ (∂*pCO*_2_/*pCO*_2_) over the fractional change in DIC (∂*DIC/DIC*) at a given temperature, salinity and TAlk (Equation (12)) [60,61]. In other words, RF quantifies the ocean’s sensitivity to an increase in atmospheric CO_2_, i.e.

(12) \begin{equation*} \mathit {RF}=\frac{ {\partial {p\rm CO}_2/{p\rm CO}_2}}{\mathit {\partial DIC/ DIC}}. \end{equation*}

In a simplified way and as an approximation, ∂*DIC* equals δDIC^*^, which is solely achieved through sea–air CO_2_ exchange, implying that ∂*p*CO_2_ may represent the sea–air Δ*p*CO_2_ (defined as the difference in *p*CO_2_ between the sea and the air, or *p*CO_2_*sea*_ − *p*CO_2_*air*_). Given an initial balance of CO_2_ between the seawater and the atmosphere, the sea–air Δ*p*CO_2_ is obtained by

(13) \begin{eqnarray*} \Delta {p\rm CO}_2&=& \mathit {RF}\times {p\rm CO}_{2\_ \mathit {air}}\times \frac{\delta {\mathit {DIC}}^{\ast }}{\mathit {DIC}}\nonumber\\ & =& \mathit {RF}\times {p\rm CO}_{2\_ \mathit {air}}\times \frac{\Delta {\mathit {DIC}}-6.6\Delta {\mathit {NO}}_3}{\mathit {DIC}}, \nonumber\\ \end{eqnarray*}

(14) \begin{eqnarray*} \Delta {{p\rm CO}}_2&=& \mathit {RF}\times {{p\rm CO}}_{2\_ \mathit {air}}\times \frac{\delta {\mathit {DIC}}^{\ast }}{\mathit {DIC}}\nonumber\\ &=& {\mathit {RF}}\times {{p\rm CO}}_{2\_ \mathit {air}}\times \frac{\Delta {\mathit {DIC}}-106\Delta {\mathit {PO}}_4}{\mathit {DIC}}.\nonumber\\ \end{eqnarray*}

The surface *p*CO_2_ in the seawater body can thus be predicted as

(15) $$\begin{eqnarray*}
{{p\rm CO}}_{2\_ \mathit {sea}\_ \mathit {pred}}={{p\rm CO}}_{2\_ \mathit {air}}+\Delta {{p\rm CO}}_2.
\end{eqnarray*}$$

## Supplementary Material

nwz105_Supplemental_File
