# Supplementary material for: Diagnosis of CO2 dynamics and fluxes in global coastal oceans
Source: Natl Sci Rev. 2019 Aug 2;7(4):786–97. doi: 10.1093/nsr/nwz105 (PMC8288922; doi:10.1093/nsr/nwz105)
Supplement: nwz105_Supplemental_File [file nwz105_supplemental_file.doc]

**Supplementary Information for**

**Diagnosis of CO_2_ dynamics and fluxes in global coastal oceans**

Zhimian Cao, Wei Yang, Yangyang Zhao, Xianghui Guo, Zhiqiang Yin, Chuanjun Du, Huade Zhao, and Minhan Dai*

State Key Laboratory of Marine Environmental Science and College of Ocean and Earth Sciences, Xiamen University, Xiamen 361102, China

*Corresponding author:

Dr. Minhan Dai

Phone: +86-592-2182132

Fax: +86-592-2184101

E-mail: mdai@xmu.edu.cn

**1 Supplementary Text**

**1.1 Latitudinal distribution of global coastal *p*CO_2_ at 50 and 100 km offshore**

While sea surface temperature (SST) clearly shows high values at low latitudes and low values at high latitudes, partial pressure of CO_2_ (*p*CO_2_) has a much less defined pattern with significant seasonal variations. It shows generally low values at mid and high latitudes and slightly high values in tropical zones in spring, with the highest values ranging around 700 and 530 μatm, respectively, at ~19°N located 50 and 100 km off the coast of West Africa (Supplementary Figs. S1e and S2e). In summer, *p*CO_2_ exhibits low values at high latitudes and relatively high values at low latitudes, with distinguished values of ~700 μatm in the Arabian Sea at ~18°N and ~640 μatm at ~19°N off the coast of West Africa (100 km offshore; Supplementary Fig. S2f). Some extremely high values are observed in boreal mid-latitude zones 50 km offshore. Here *p*CO_2_ is close to ~700 μatm in the Baltic Sea at ~55°N, while *p*CO_2_ ranges from ~600 to >800 μatm at ~38°N off the California-Oregon coast (Supplementary Fig. S1f). In autumn, the latitudinal pattern of *p*CO_2_ is not so clear, but in the Baltic Sea *p*CO_2_ is even higher than in summer, approaching ~1000 μatm (Supplementary Figs. S1g and S2g). In winter, the *p*CO_2_ values at mid latitudes are slightly lower compared to other latitudes, with high values of >600 (50 km offshore) and >700 (100 km offshore) μatm in the Baltic Sea and values close to 700 (50 km offshore) and 550 (100 km offshore) μatm at ~21°S off the Peru coast (Supplementary Figs. S1h and S2h). The sea-air Δ*p*CO_2_ (defined as the *p*CO_2_ difference between the sea and the air, or *p*CO2_sea−*p*CO2_air) at both 50 and 100 km offshore during each season (Supplementary Figs. S1i-l and S2i-l) shows nearly the same latitudinal pattern as sea surface *p*CO_2_.

With respect to the inter-seasonal *p*CO_2_ changes (δ*p*CO_2_), we infer that different latitudinal bands vary in their main controls. During the seasonal changes from winter to spring (Supplementary Figs. S1m and S2m) and from summer to autumn (Supplementary Figs. S1o and S2o), non-thermal factors prevail in nearly all latitudinal bands at both 50 and 100 km offshore, while from spring to summer (Supplementary Figs. S1n and S2n), temperature mainly explains the *p*CO_2_ variations in bands from 20°N-45°N and non-thermal factors control the other bands. From autumn to winter (Supplementary Figs. S1p and S2p), non-thermal controls prevail for bands >45°N or >40°S, while temperature is a major control for *p*CO_2_ changes in bands from 45°N to 40°S at both 50 and 100 km offshore.

**1.2 Seasonal** **variations of *p*CO_2_ in the South China Sea and the Arabian Sea**

*1.2.1 p*CO_2_ *data in the South China Sea*

Sampling was conducted onboard the *R/V Dongfanghong II* during four cruises to the South China Sea in spring (April 29-June 10) 2011, summer (July 17-September 1) 2009, autumn (October 20-December 11) 2010, and winter (December 23, 2009-February 6, 2010) 2009. Surface waters for underway *p*CO_2_ measurements were pumped continuously from ~5 m depth into a shipboard laboratory, while the bow intake from which atmospheric air was pumped for *p*CO_2_ measurements was installed at ~15 m above the sea surface to avoid contamination from the ship. We selected *p*CO_2_ data collected from an area of 18°N-20°N and 114°E-118°E for the seasonal variation analysis (Supplementary Fig. S4).

Atmospheric and surface water *p*CO_2_ was determined using an automated underway system developed by the CO_2_ Group of NOAA’s Atlantic Oceanographic and Meteorological Lab (GO-8050, General Oceanics Inc) [1]. The CO_2_ mole fraction in dry air (*x*CO_2_) was detected continuously using a Li-Cor 7000 non-dispersive infrared (NDIR) spectrometer coupled to a gas-water equilibrator. The NDIR spectrometer was calibrated against four CO_2_ gas standards with *x*CO_2_ values of 201.7, 399.5, 597.8, and 785.8 μmol mol^-1^. The *p*CO_2_ data were finally obtained according to the procedures previously described in our work [2], with an overall uncertainty of <1% [3].

*1.2.2 p*CO_2_ *data in the Arabian Sea*

Five cruises to the Arabian Sea were conducted in 1995 aboard the *R/V Thomas G. Thompson* as part of the US Joint Global Ocean Flux Study (JGOFS) (http://usjgofs.whoi.edu/jg/dir/jgofs/) [4]. We selected the underway *p*CO_2_ data collected during these cruises, which are ttn-043 during the Northeast Monsoon season (winter; January 8-February 1), ttn-045 during the Spring Intermonsoon season (spring; March 14-April 8), ttn-049 during the Southwest Monsoon season (summer; July 18-August 13), ttn-053 during the Autumn Intermonsoon season (autumn; October 29-November 25), and ttn-054 during the Northeast Monsoon season (winter; November 30-December 26). We divided the study area into four domains to quantify the inter-seasonal *p*CO_2_ changes because of large spatial variability in sea surface *p*CO_2_ during a single season (Supplementary Fig. S5).

*1.2.3 Analysis of inter-seasonal pCO_2_ changes*

We applied the same calculations (Eqs. (1)-(4); see ‘Methods’ section) to analyze inter-seasonal *p*CO_2_ variations in the South China Sea and the Arabian Sea. The only difference from the analysis of global coastal *p*CO_2_ data is that *p*CO2_X and *p*CO2_Y in Eq. (1) are the averages of all field-observed sea surface *p*CO_2_ during X season and Y season, respectively, in the two marginal seas.

**1.3 OceMar case I: South China Sea basin**

*1.3.1 South China Sea background*

The South China Sea is the largest marginal sea of the Pacific Ocean with a surface area of 3.5×10^6^ km^2^ and an average depth of 1350 m. It is semi-enclosed and has only one source of deep water from the adjacent western North Pacific through the 2200 m deep Luzon Strait [5]. The deeper part of the South China Sea is confined to a bowl-shaped trench with the maximum depth around 5560 m, forming a completely isolated basin below 2400 m. An overflow, driven by a persistent baroclinic pressure gradient, makes the colder and denser Pacific water sink to the deep South China Sea after it crosses the Luzon Strait [6]. Given the fast vertical replenishment, the deep South China Sea water must rise to the upper layer through strong diapycnal mixing [7].

*1.3.2 Cruises and sampling*

Sampling was conducted onboard the *R/V Dongfanghong II* during an autumn (November 9-22, 2010) and a summer (May 28-July 14, 2014) cruise to the South China Sea. During both cruises, sea surface and atmospheric *p*CO_2_ samples were collected continuously using the same method described in subsection 1.2.1, while high spatial resolution measurements of the carbonate and nutrient chemistry through the entire water column were performed at stations deeper than 2500 m in the basin area (Supplementary Fig. S6). Seawater profile samples were collected with Niskin bottles attached to a Rosette sampler. Samples for dissolved inorganic carbon (DIC) and total alkalinity (TAlk) analyses were stored in 40 mL borosilicate glass vials and 125 mL polyethylene bottles, respectively. Both DIC and TAlk samples were poisoned using an HgCl_2_-saturated solution upon sample collection, with 40 μL for DIC and 50 μL for TAlk. The majority of the nutrient samples were analyzed onboard immediately while surface water samples with extremely low nitrate (NO_3_) and phosphate (PO_4_) concentrations were stored in acid pre-cleaned polyethylene bottles at −20°C for analysis onshore.

Surface seawater temperature and conductivity were measured continuously using an Idronaut Multiparameter “Flow Through” CTD recorder. Depth profiles of temperature and salinity were determined shipboard with a calibrated SBE-19-plus CTD recorder (Sea-Bird Electronics Inc.) attached to the Rosette sampler.

*1.3.3 Analyses of carbonate system and nutrient parameters*

DIC samples were analyzed onboard within two days of sampling, while TAlk samples were analyzed within a few weeks after the cruise. DIC was determined by acidification of a 0.5 mL water sample and subsequent quantification of CO_2_ with a Li-Cor 7000 NDIR spectrometer. TAlk was determined using Gran titration on a 25 mL sample with a Kloehn digital syringe pump. Each method has a precision of ±2 μmol kg^-1^. Both DIC and TAlk were calibrated against the certified reference material provided by A. Dickson of the Scripps Institution of Oceanography [8].

NO_3_ and PO_4_ concentration data were mainly collected onboard using a Technicon AA3 Auto-Analyzer (Bran-Lube, GmbH Co). PO_4_ was measured according to classical colorimetric methods, while NO_3_ was analyzed using the on-line copper-cadmium column reduction method. The precision for PO_4_ and NO_3_ was within ±2% and ±1%, respectively [9]. In addition, NO_3_ and PO_4_ at nM levels in surface waters were measured with a precision of better than ±5% [10,11].

*1.3.4 Water mass mixing above the surface mixed layer*

The statistically significant positive TAlk-salinity relationships suggest that waters in the surface mixed layer of the South China Sea basin (<50 m in both autumn 2010 and summer 2014) originate from two-endmember mixing between waters immediately below the surface mixed layer and rain water, the latter indicated by the near-zero intercepts. Normalized TAlk (NTAlk, normalized to a salinity of 34.0) generally shows an invariable trend with increasing salinity, which also points to the presence of a rain water endmember with zero solutes during both seasons (Supplementary Fig. S7). The conservative-mixing-induced DIC, NO_3_, and PO_4_ (Eqs. (7)-(9); see ‘Methods’ section) values were thus calculated as:

$X^{cons}=\frac{Sal^{meas}}{Sal^{ref}}\times X^{ref}$ (S1)

X represents DIC, NO_3_, or PO_4_, while Sal^meas^ is the CTD recorder-measured salinity. Sal^ref^ and X^ref^ are the reference salinity and concentration of DIC, NO_3_, or PO_4_ in waters immediately below the surface mixed layer, respectively. The sub-mixed layer endmember is based on average values of 50-75 m samples for the cruise in autumn 2010 and of 25-50 m samples for the cruise in summer 2014.

**1.4 OceMar case II: Arabian Sea basin**

*1.4.1 Arabian Sea background*

The Arabian Sea is a large marginal sea of the Indian Ocean covering a surface area of 3.9×10^6^ km^2^ and an average depth of 2730 m. It is strongly influenced by the Asian monsoon, experiencing a biannual monsoonal reversal of surface winds that blow from the southwest in summer and from the northeast in winter, which drives seasonal variations in surface circulation [12,13]. During the southwest monsoon season, a northeast-flowing coastal boundary current, the Somali Current, forms a series of anticyclonic gyres off the coasts of Somali, Yemen, and Oman. Coastal upwelling frequently occurs in this season, leading to high primary production. During the northeast monsoon season, however, the surface Somali Current and the West India Coastal Current weaken and reverse to flow southward and northward, respectively [13,14]. The Arabian Sea basin is a potential OceMar since it exchanges water with the Indian Ocean in at least two-dimensions. Deep waters from the Indian Ocean intrude into the continental margins via horizontal advection, and are then transported upward via vertical mixing and upwelling in the Arabian Sea interior [15].

*1.4.2 Cruises and data*

We selected carbonate system and nutrient data collected during five Arabian Sea cruises conducted in 1995 (http://usjgofs.whoi.edu/jg/dir/jgofs/; Supplementary Fig. S9) to characterize *p*CO_2_ in the surface mixed layer of the Arabian Sea basin. Cruises ttn-043, ttn-045, ttn-049, ttn-053, and ttn-054 were conducted during the Northeast Monsoon season (January 8-February 1; winter), Spring Intermonsoon season (March 14-April 8; spring), Southwest Monsoon season (July 18-August 13; summer), Autumn Intermonsoon season (October 29-November 25; autumn), and Northeast Monsoon season (November 30-December 26; winter).

*1.4.3 Water mass mixing above the surface mixed layer of the Arabian Sea basin*

TAlk and salinity in the surface mixed layer (upper 75 m for cruise ttn-045 and upper 100 m for cruises ttn-043, ttn-049, ttn-053, and ttn-054) of the Arabian Sea basin show statistically significant positive relationships in all seasons, indicating an overall two-endmember mixing scheme for each season (Supplementary Fig. S10). For cruises ttn-045, ttn-053, and ttn-054, the intercepts of the linear regression lines between TAlk and salinity are around or near zero, suggesting that waters in the surface mixed layer of the corresponding seasons originated from subsurface waters that were subsequently diluted by rain water with zero solutes. NTAlk (normalized to a salinity of 36.5) remains constant with salinity which also points to a rain water endmember (Supplementary Fig. S10b,d,e). For cruises ttn-043 and ttn-049, however, the intercepts of the linear regression lines are ~160 and ~250 μmol kg^-1^, respectively, suggesting a nonzero-solute freshwater endmember at zero salinity. Such an endmember, resulting in a declining trend of NTAlk with increasing salinity (Supplementary Fig. S10a,c), influenced the carbon and nutrients in the surface waters of the Arabian Sea during the northeast and southwest monsoon seasons. In this case, the conservative-mixing-induced values were estimated as:

$X^{cons}=\frac{Sal^{meas}}{Sal^{ref}}\times(X^{ref}-X^{eff})+X^{eff}$ (S2)

Sal^ref^ and X^ref^, as used in Eq. (S1), are the reference salinity and concentration of a given parameter in the water mass endmember immediately below the surface mixed layer, calculated as the average of all 100 m samples for cruises ttn-043, ttn-049, ttn-053, and ttn-054 and of all 75 m samples for cruise ttn-045. X^eff^ denotes the effective concentration of DIC, NO_3_, or PO_4_ that finally enters the Arabian Sea from the freshwater endmember inputs.

The Euphrates and Tigris Rivers are two major rivers that largely contribute to the freshwater discharge to the Arabian Sea. TAlk, NO_3_, and PO_4_ concentrations in the mainstream of both the Euphrates and Tigris Rivers [16,17] are ~2400, ~30, and ~3 μmol kg^-1^, respectively. Since bicarbonate dominates inorganic carbon species other than CO_2_ in a number of rivers, e.g., the Mississippi and Columbia Rivers [18,19], DIC concentrations in the Euphrates and Tigris Rivers are assumed to be numerically similar to TAlk, around ~2400 μmol/kg^-1^.

Assuming that the biological consumption of DIC and PO_4_ in the river plume followed Redfield stoichiometry [20], DIC removal is estimated to be ~300 µmol kg^−1^ (approximately 3×10^6^), while PO_4_ was rapidly consumed along the pathway of the river plume and generally depleted in areas beyond the plume. As a consequence, the complete values of DIC^eff^ and PO_4_^eff^ in the surface waters of the Euphrates and Tigris Rivers would be ~2100 µmol kg^−1^ and ~0 µmol kg^-1^.

If the nonzero-solute freshwater endmember is a mixture of river and rain water with zero solutes, the intercept values of ~160 (Supplementary Fig. S10a) and ~250 (Supplementary Fig. S10c) derived from the TAlk-salinity regressions would indicate that the river fractions were ~6.5 and ~10.4% (approximately 160/2400 and 250/2400 taking ~2400 μmol kg^-1^ as the TAlk endmember value of the Euphrates and Tigris Rivers). DIC^eff^ from the freshwater input is thus estimated to be ~130 μmol kg^-1^ (approximately 2100×6.5%) and ~220 µmol kg^-1^ (approximately 2100×10.4%) for cruises ttn-043 and ttn-049, respectively. PO_4_^eff^ in any combined freshwater endmember is zero.

**1.5 RiOMar case I: Pearl River Plume**

*1.5.1 Northern South China Sea shelf background*

The northern South China Sea shelf, stretching from the northeast to the southwest of mainland China, has an offshore extension of 150 to 300 km, and covers a surface area of about 1.2×10^6^ km^2^. Coastal upwelling frequently occurs along the coast, driven by the southwest monsoon in summer. Strong variability of the upwelling current exists both in alongshore and cross-shore directions due to the highly variable shelf topography [21]. The northern South China Sea shelf also features prevalent river plume water originating from the Pearl River, a globally major river system with a water discharge of 3.26×10^11^ m^3^ yr^-1^. It is noted that ~80% of the discharge takes place during the wet season (April-September) [8]. Thus, the northern South China Sea shelf in summer represents an important and unique RiOMar system under the co-influence of the river plume and coastal upwelling [22].

*1.5.2 Cruises and data*

Two mapping cruises onboard the *R/Vs Shiyan III* and *Haike 86* were conducted on the northern South China Sea shelf in summer 2008 (June 30-July 8) and 2016 (July 20-31), respectively. Cross-shelf transects covering the region from the Pearl River estuary to east of the Taiwan Shoals were intensively investigated (Supplementary Fig. S12). As a consequence of the continuous heavy rain in the upstream Pearl River, an abundant river water input generated patches of plume water that spread eastward over the northern South China Sea shelf during the sampling periods of both 2008 and 2016. During both cruises, high spatial resolution data of the carbonate system and nutrients throughout the water column were collected, which were used in this study to evaluate the CO_2_ source/sink nature of the Pearl River plume waters (salinity <33.0) on the northern South China Sea shelf.

*1.5.3 ΔDIC, ΔNO_3_, and ΔPO_4_ beyond a three-endmember mixing control on the northern South China Sea shelf*

In the present Pearl River plume case, the water mass mixing scheme is complex because the shelf system is under the co-influence of both the river plume and coastal upwelling. A three-endmember mixing model was adopted to establish the physical dynamics and biogeochemistry of the northern South China Sea shelf [8], upon which our diagnostic framework is applied as for the case of OceMars. The three-endmember mixing scheme was identified in the upper 100 m of the water column on the northern South China Sea shelf between plume water, offshore subsurface water and offshore surface water as suggested by the temperature-salinity diagram (Supplementary Fig. S13). In this context, the thermohaline properties of any sample are the sum of the contribution of the three endmembers, which can be calculated as:

$F_{p}+F_{u}+F_{s}=1$ (S3)

$\theta_{p}F_{p}+\theta_{u}F_{u}+\theta_{s}F_{s}=\theta^{meas}$ (S4)

$S_{p}F_{p}+S_{u}F_{u}+S_{s}F_{s}=S^{meas}$ (S5)

F_p_, F_u_, and F_s_ are the respective fractions of plume water, offshore subsurface water, and offshore surface water in the water sample. θ_p_, θ_u_, and θ_s_ are the potential temperatures of the three endmember waters, while S_p_, S_u_, and S_s_ denote the salinities of the endmembers. θ^meas^ and S^meas^ are the field-observed potential temperature and salinity of the water sample.

The conservative-mixing-induced DIC, NO_3_, and PO_4_ concentrations of the three endmembers can thus be calculated as:

$DIC^{cons}=DIC_{p}F_{p}+DIC_{u}F_{u}+DIC_{s}F_{s}$ (S6)

$NO_{3}^{cons}=NO_{3p}F_{p}+NO_{3u}F_{u}+NO_{3s}F_{s}$ (S7)

$PO_{4}^{cons}=PO_{4p}F_{p}+PO_{4u}F_{u}+PO_{4s}F_{s}$ (S8)

DIC_p_/NO_3p_/PO_4p_, DIC_u_/NO_3u_/PO_4u_, and DIC_s_/NO_3s_/PO_4s_ are the DIC/NO_3_/PO_4_ concentrations of the three endmember waters. The difference between the conservative and observed concentration (ΔDIC/ΔNO_3_/ΔPO_4_ in Eqs. (7)-(9); see ‘Methods’ section), that is not accounted for by the model, represents the influence of other processes with positive values indicating removal and negative ones suggesting addition of DIC and nutrients.

**1.6 RiOMar case II: Amazon River plume**

*1.6.1 Amazon River plume background*

The Amazon River, the largest river in the world, delivers ~16% of global riverine input to the ocean [23]. With a mean annual transport [24] of 1.8-2.8×10^5^ m^3^ s^-1^, the Amazon River generates an offshore plume extending far beyond the river mouth to the oligotrophic western tropical North Atlantic. Due to the dominance of wind forcing and the northwestward North Brazil Current over weak low-latitude Coriolis acceleration, the plume water shifts to an along-shore northwesterly transport during boreal winter and spring [25] and subsequently detaches from the coast anticyclonically and retroflects northeast into the western tropical North Atlantic [25,26]. During peak autumn surface flow of the North Equatorial Countercurrent, a large volume of low-inorganic carbon, high-chlorophyll plume water can reach as far as 3000 km east to 25°W, covering up to ~2×10^6^ km^2^ and accounting for >20% of the western tropical North Atlantic [27-29].

*1.6.2 Cruise and data*

Data of the carbonate system and nutrients were collected during the spring cruise aboard the *R/V Seward Johnson I* to the western tropical North Atlantic during April 19-May 20, 2003 (Supplementary Fig. S15) [30]. DIC and TAlk data were derived from the datasets in CARINA_v1.2 (Cruise ID: 90), while nutrient, surface mixed layer depth, and N_2_ fixation rate data from the same cruise were obtained in the study of Subramaniam et al. (2008) [31]. Stations on the continental shelf influenced by the Amazon River outer plume were identified by low surface salinities of 30.0-35.0. The TAlk value of the river water endmember at zero salinity was obtained by extrapolation of the linear regression between TAlk and salinity (Supplementary Fig. S16), assuming TAlk is overall conservative during water mass mixing despite minor changes induced by biological modulation. DIC at the mouth of the Amazon River estuary was expected to vary with TAlk at a fixed ratio (TAlk=0.82×DIC) [32]. Nutrient values of the river water endmember were derived from a previous study [33]. Atmospheric *p*CO_2_ was ~370 μatm at Ragged Point, Barbados, obtained from the NOAA Earth System Research Laboratory.

*1.6.3 NO_3_ and PO_4_ deficit beyond a two-endmember mixing control in the Amazon River outer plume*

Despite complex interactions between various water masses in the western tropical North Atlantic, a two-endmember mixing scheme was identified for the Amazon River outer plume between the river water and the offshore surface water as suggested by the statistically significant positive relationship between TAlk and salinity (Supplementary Fig. S16). In this context, the thermohaline properties of any sample are the sum of the contribution of the two endmembers, which can be calculated as:

$F_{\text{r}}+F_{s}=1$ (S9)

$S_{r}F_{r}+S_{s}F_{s}=S^{meas}$ (S10)

F_r_ and F_s_ are the respective fractions of river water and offshore surface water in the water sample, while S_r_ and S_s_ represent the salinities of the endmembers. S^meas^ is the in-situ salinity of the water sample.

The conservative-mixing-induced DIC, NO_3_, and PO_4_ concentrations of the water sample can thus be calculated as:

$DIC^{cons}=DIC_{r}F_{r}+DIC_{s}F_{s}$ (S11)

$NO_{3}^{cons}=NO_{3r}F_{r}+NO_{3s}F_{s}$ (S12)

$PO_{4}^{cons}=PO_{4r}F_{r}+PO_{4s}F_{s}$ (S13)

DIC_r_/NO_3r_/PO_4r_ and DIC_s_/NO_3s_/PO_4s_ are the DIC/NO_3_/PO_4_ concentrations of the two endmember waters, river water and offshore surface water, respectively. The difference between the conservative and observed concentrations (ΔDIC/ΔNO_3_/ΔPO_4_ in Eqs. (7)-(9); see ‘Methods’ section) that is not accounted for by the model represents the influence of other processes, with positive values indicating removal and negative ones suggesting addition of DIC and nutrients.

Due to additional nutrient supplies from N_2_ fixation and DOP, ΔNO_3_ and ΔPO_4_ only denote the apparent consumption of NO_3_ and PO_4_. The actual removal of NO_3_ and PO_4_ can be back-calculated based on the deduction of the sea-air exchange-induced DIC variations (δDIC^*^ in Eqs. (10) and (11); see ‘Methods’ section) from ΔDIC. Thus, the deficit of NO_3_ and PO_4_ can be estimated as the difference of the actual removal and apparent consumption.

**2 Supplementary Figures and Captions**

**
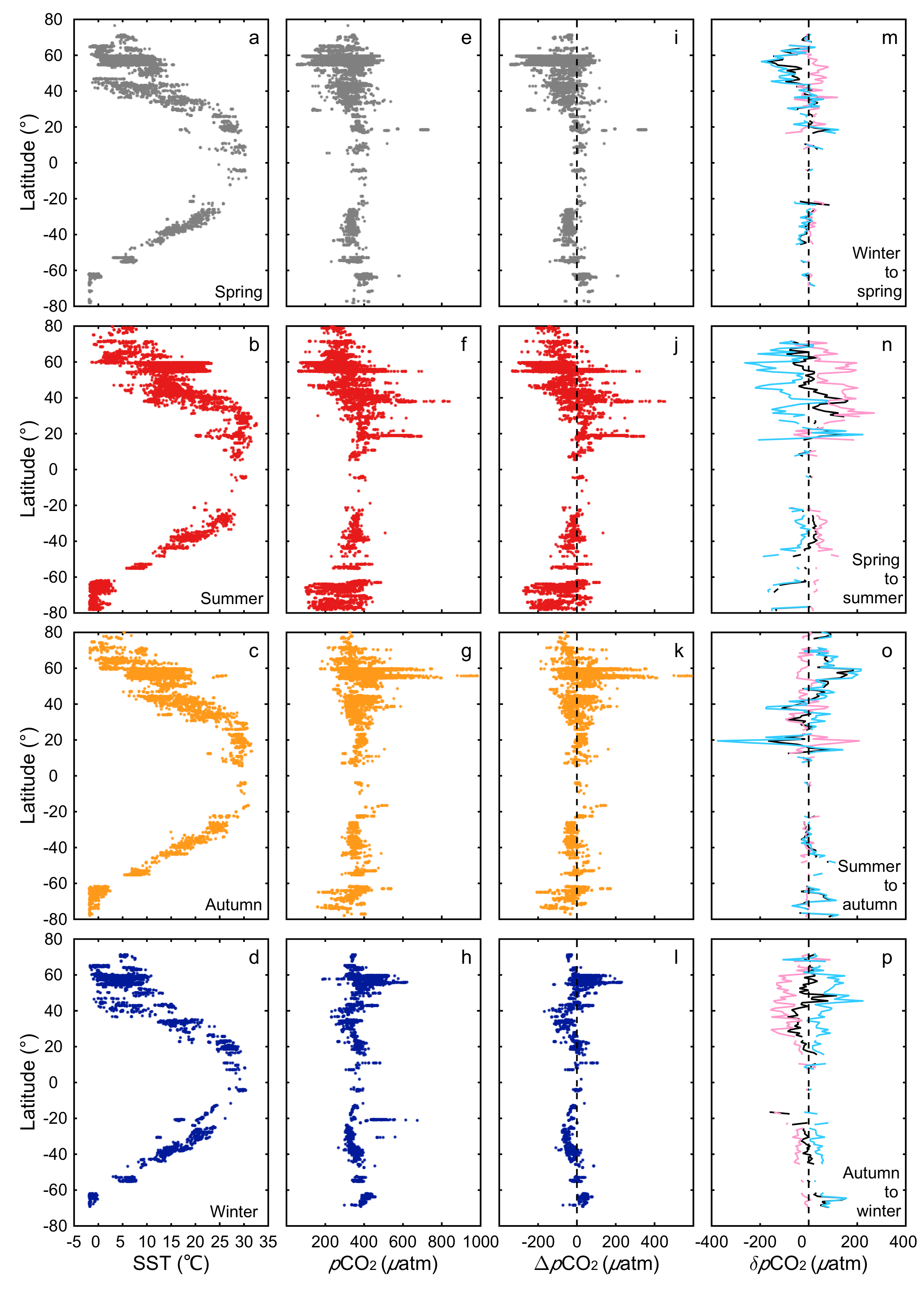
**

**Figure S1 | Evidence for non-thermal controls on partial pressure of CO_2_ (*p*CO_2_) in global coastal oceans.** Data were selected at a distance of 50 km from the major land masses. **a**-**d**, Latitudinal distribution of sea surface temperature (SST) in various seasons. **e**-**h**, Latitudinal distribution of surface *p*CO_2_ in various seasons. **i*-*l**, Latitudinal distribution of sea-air Δ*p*CO_2_ (defined as the difference of *p*CO_2_ between the sea and the air, or *p*CO2_sea−*p*CO2_air) in various seasons. While SST clearly shows high values at low latitudes and low values at high latitudes, *p*CO_2_ and sea-air Δ*p*CO_2_ display a mismatch to SST, having a much less defined pattern during each season. **m**-**p**, Change in *p*CO_2_ between two consecutive seasons (δ*p*CO_2_), averaged over a 1°-latitude band; **Black**, pink, and blue lines indicate δ*p*CO_2_ from **field observations**, solely due to temperature variations, and controlled by other factors such as mixing and biogeochemical processes, respectively (Eqs. (1)-(4); see ‘Methods’ section). Although different latitudinal bands have different main controls of δ*p*CO_2_ during each seasonal transition, the inter-seasonal changes of global coastal *p*CO_2_ are overall more determined by non-thermal factors than temperature.


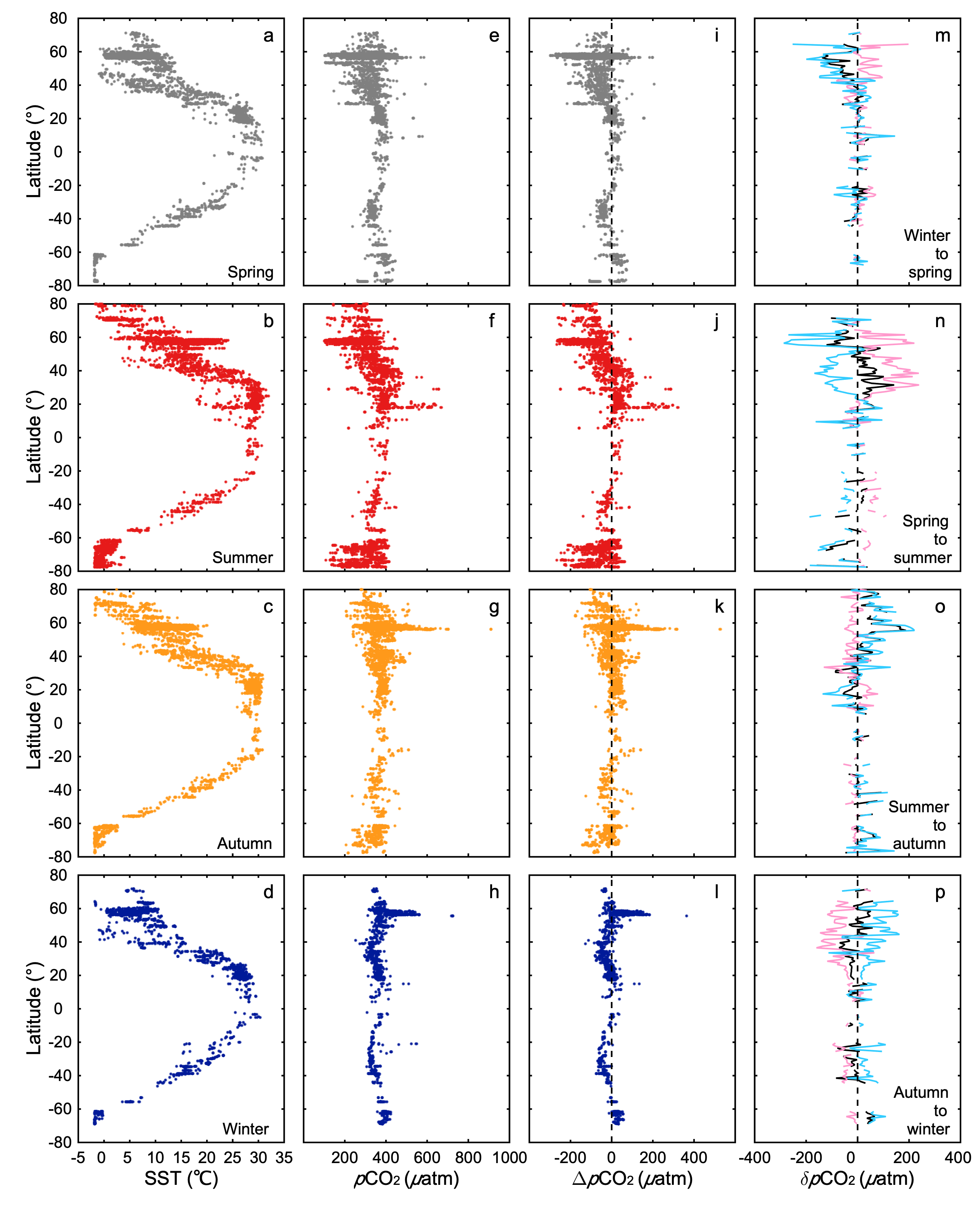


**Figure S2 | Evidence for non-thermal controls on partial pressure of CO_2_ (*p*CO_2_) in global coastal oceans.** Data were selected at a distance of 100 km from the major land masses. **a**-**d**, Latitudinal distribution of sea surface temperature (SST) in various seasons. **e**-**h**, Latitudinal distribution of surface *p*CO_2_ in various seasons. **i*-*l**, Latitudinal distribution of sea-air Δ*p*CO_2_ (defined as the difference of *p*CO_2_ between the sea and the air, or *p*CO2_sea−*p*CO2_air) in various seasons. While SST clearly shows high values at low latitudes and low values at high latitudes, *p*CO_2_ and sea-air Δ*p*CO_2_ display a mismatch to SST, having a much less defined pattern during each season. **m**-**p**, Change in *p*CO_2_ between two consecutive seasons (δ*p*CO_2_), averaged over a 1°-latitude band; **Black**, pink, and blue lines indicate δ*p*CO_2_ from **field observations**, solely due to temperature variations, and controlled by other factors such as mixing and biogeochemical processes, respectively (Eqs. (1)-(4); see ‘Methods’ section). Although different latitudinal bands have different main controls of δ*p*CO_2_ during each seasonal transition, the inter-seasonal changes of global coastal *p*CO_2_ are overall more determined by non-thermal factors than temperature.

**Figure S3 | Global map showing the location of SOCAT v3.0 *p*CO_2_ sampling stations.** All stations are with a distance of (a) 50 km and (b) 100 km to major land masses. The insert in both panels is a zoom-in of the west coast of US and Canada. The color bar indicates water depth.

**Figure S4 | Surface distribution of *p*CO_2_ in the deep basin of the northern South China Sea. a**, Spring (April 29-June 10) 2011. **b**, Summer (July 17-September 1) 2009. **c**, Autumn (October 20-December 11) 2010. **d**, Winter (December 23, 2009-February 6, 2010) 2009. The color bar indicates the *p*CO_2_ level (in μatm), which is higher in spring and summer than in autumn and winter.

**Figure S5 | Surface distribution of *p*CO_2_ in the Arabian Sea in 1995. a**, Cruise ttn-043 in winter (January 8-February 1). **b**, Cruise ttn-045 in spring (March 14-April 8). **c**, Cruise ttn-049 in summer (July 18-August 13). **d**, Cruise ttn-053 in autumn (October 29-November 25). **e**, Cruise ttn-054 in winter (November 30-December 26). The color bar indicates the *p*CO_2_ level (in μatm), which shows large spatial variability during each cruise. In order to quantify the seasonal change of *p*CO_2_, we divided the study area into four domains: (1) Coastal upwelling; (2) Indian shelf; (3) Findlater Jet axis; (4) Southeastern stations.

**Figure S6 | Map of the South China Sea showing the location of sampling stations in the deep basin.** Pink triangles: autumn (November 9-22) 2010. Blue triangles: summer (May 28-July 14) 2014. The color bar indicates water depth.

**Figure S7 |** **Two-endmember mixing scheme in the upper 50 m of the South China Sea basin reflected by the relationship between total alkalinity (TAlk) and salinity.** **a**, Autumn 2010. **b**, Summer 2014. The black lines as well as the equations indicate the linear regression analyses of the TAlk-salinity relationship. The statistically significant positive relationship during both seasons suggests that waters in the surface mixed layer originate from two-endmember mixing between waters immediately below the surface mixed layer and rain water, the latter which is indicated by the near zero intercepts. Normalized TAlk (NTAlk, normalized to a salinity of 34.0) generally shows an invariable trend with increasing salinity, also pointing to the presence of a rain water endmember with zero solutes during both seasons.

**Figure S8 | Application of our semi-analytical framework to the South China Sea basin as an ocean-dominated margin (OceMar). a**,**b**, Autumn 2010. **c**,**d**, Summer 2014. “ΔDIC−106ΔPO_4_” (red circles in **a** and **c**) denotes the consumption of DIC relative to PO_4_ (Eq. (7) in Box 1) based on the Redfield C/P consumption ratio of 106 [20]. “*p*CO_2_ estimated” (blue triangles in **a** and **c**) denotes the predicted *p*CO_2_ using our semi-analytical diagnostic framework (Eqs. (7)-(15); see ‘Methods’ section), while “*p*CO_2sys_” (**black** circles in **b** and **d**) denotes the corresponding calculated *p*CO_2_ by applying TAlk and DIC data to the CO2SYS program [34]. “*p*CO_2_ underway” (gray triangles in **b** and **d**) denotes the field underway observations of sea surface *p*CO_2_. The dashed gray line in **b** and **d** indicates the average field-observed atmospheric *p*CO_2_ of ~380 μatm during both seasons.

**Figure S9** **| Map of the Arabian Sea showing the location of sampling stations** **investigated in 1995.** **a**, Cruise ttn-043 in winter (January 8-February 1). **b**, Cruise ttn-045 in spring (March 14-April 8). **c**, Cruise ttn-049 in summer (July 18-August 13). **d**, Cruise ttn-053 in autumn (October 29-November 25). **e**, Cruise ttn-054 in winter (November 30-December 26).

**Figure S10 |** **Two-endmember mixing scheme in the upper water column of the Arabian Sea basin reflected by the relationship between total alkalinity (TAlk) and salinity.** **a**, Cruise ttn-043 (upper 100 m). **b**, Cruise ttn-045 (upper 75 m). **c**, Cruise ttn-049 (upper 100 m). **d**, Cruise ttn-053 (upper 100 m). **e**, Cruise ttn-054 (upper 100 m). The black lines as well as the equations indicate the linear regression analyses of the TAlk-salinity relationship. The statistically significant positive relationship for each season suggests a two-endmember mixing scheme in the surface mixed layer constrained by the subsurface waters and rain water with zero solutes (indicated by the near zero intercepts in **b**, **d**, and **e**) or nonzero-solute freshwater (indicated by the nonzero intercepts in **a** and **c**). In **b**, **d**, and **e**, normalized TAlk (NTAlk, normalized to a salinity of 36.5) remains constant with salinity pointing to a rain water endmember. In **a** and **c**, however, NTAlk shows a declining trend with increasing salinity, resulting from a nonzero-solute freshwater endmember.

**Figure S11 |** **Application of our semi-analytical framework to the Arabian Sea basin as an OceMar. A a**,**b**, Cruise ttn-043. **c**,**d**, Cruise ttn-045. **e**,**f**, Cruise ttn-049. **g**,**h**, Cruise ttn-053. **i**,**j**, Cruise ttn-054. In the left panels, “ΔDIC−106ΔPO_4_” (red circles) denotes the consumption of DIC relative to PO_4_ (Eq. (7) in Box 1) based on the Redfield C/P consumption ratio of 106 [20]. “*p*CO_2_ estimated” (blue triangles) denotes the predicted *p*CO_2_ using our semi-analytical diagnostic framework (Eqs. (7)-(15); see ‘Methods’ section). In the right panels, “*p*CO_2sys_” (**black** circles) denotes the corresponding calculated *p*CO_2_ by applying TAlk and DIC data to the CO2SYS program [34], while “*p*CO_2_ underway” (gray triangles) denotes the field underway observations of sea surface *p*CO_2_. The dashed gray line indicates the field-observed atmospheric *p*CO_2_, which is on average 356 µatm for cruise ttn-043, 353 µatm for cruise ttn-045, 341 µatm for cruise ttn-049, 337 µatm for cruise ttn-053, and 345 µatm for cruise ttn-054.

**Figure S12 | Map of the northern South China Sea showing the location of sampling stations on the shelf.** Blue crosses: summer (June 30-July 8) 2008. Red circles: summer (July 20-31) 2016. The color bar indicates water depth.

**Figure S13 | Three-endmember mixing scheme for the upper 100 m of the water column on the northern South China Sea shelf, reflected by the relationship between potential temperature (PT) and salinity.** **a**, Summer 2008. **b**, Summer 2016. The inset in **a** shows a general schematic of the three-endmember mixing processes observed between the plume water, offshore surface water, and offshore subsurface water during both cruises.

**Figure S14 | Application of our semi-analytical framework to the Pearl River plume as a river-dominated ocean margin (RiOMar).** **a**,**b**, Summer 2008. **c**,**d**, Summer 2016. “ΔDIC−6.6ΔNO_3_” (red circles in **a** and **c**) denotes the consumption of DIC relative to NO_3_ (Eq. (10); see ‘Methods’ section) based on the Redfield C/N consumption ratio of 6.6 [20]. “*p*CO_2_ estimated” (blue triangles in **a** and **c**) denotes the predicted *p*CO_2_ using our semi-analytical diagnostic framework (Eqs. (7)-(15); see ‘Methods’ section), while “*p*CO_2sys_” (**black** circles in **b** and **d**) denotes the corresponding calculated *p*CO_2_ by applying TAlk and DIC data to the CO2SYS program [34]. The dashed gray line in **b** and **d** indicates the average field-observed atmospheric *p*CO_2_ of ~370 and ~390 μatm, respectively, during each season.

**Figure S15 | Map of the western tropical North Atlantic showing the location of sampling stations in April 19-May 20, 2003.** The color bar indicates water depth.

**Figure S16 | Two-endmember mixing scheme in the Amazon River plume reflected by the relationship between TAlk and salinity.** The solid line as well as the equation indicates the linear regression analysis of the TAlk-Salinity relationship. The statistically significant positive relationship suggests a two-endmember mixing scheme between the river water and the offshore surface seawater.

**3 Supplementary Tables**

**Table S1 | Inter-seasonal variations of *p*CO_2_ in the South China Sea.** δ*p*CO2_total_XtoY, δ*p*CO2_temp_XtoY, and δ*p*CO2_others_XtoY denote *p*CO_2_ variations from seasons X to Y that were field-observed, solely induced by temperature, or resulted from other processes (e.g., sea-air exchange, water mass mixing, and net primary production), respectively (Eqs. (1)-(4); see ‘Methods’ section). The uncertainties, which were largely due to the spatial variability of the observations, represent one standard deviation of the *p*CO_2_ variations.

| Inter-season  from X to Y | δ*p*CO2_total_XtoY  (μatm) | δ*p*CO2_temp_XtoY  (μatm) | δ*p*CO2_others_XtoY  (μatm) |
| --- | --- | --- | --- |
| Winter to spring | 41±15 | 52±20 | −11±25 |
| Spring to summer | −6±13 | 17±11 | −23±17 |
| Summer to autumn | −24±23 | −47±23 | 23±26 |
| Autumn to winter | −11±25 | −21±29 | 10±38 |

**Table S2 | Inter-seasonal variations of *p*CO_2_ in the Arabian Sea.** δ*p*CO2_total_XtoY, δ*p*CO2_temp_XtoY, and δ*p*CO2_others_XtoY denote *p*CO_2_ variations from seasons X to Y that were field-observed, solely induced by temperature, or resulted from other processes (e.g., sea-air exchange, water mass mixing, and net primary production), respectively (Eqs. (1)-(4); see ‘Methods’ section). The study area was divided into four domains: (1) Coastal upwelling; (2) Indian shelf; (3) Findlater Jet axis; (4) Southeastern stations (Supplementary Fig. S5). The uncertainties, which were largely due to the spatial variability of the observations, represent one standard deviation of the *p*CO_2_ variations.

| Domain | Inter-season  from X to Y | δ*p*CO2_total_XtoY  (μatm) | δ*p*CO2_temp_XtoY  (μatm) | δ*p*CO2_others_XtoY  (μatm) |
| --- | --- | --- | --- | --- |
| 1 | Winter to spring | 3±19 | 27±4 | −24±19 |
|  | Spring to summer | 180±68 | −40±4 | 220±68 |
|  | Summer to autumn | −182±68 | 93±17 | −275±70 |
|  | Autumn to winter | −3±15 | −18±2 | 15±15 |
| 2 | Winter to spring | −22±11 | 7±1 | −29±11 |
|  | Spring to summer | 2±9 | 27±1 | −25±9 |
|  | Summer to autumn | 8±8 | 8±1 | 0±8 |
|  | Autumn to winter | −15±10 | −18±1 | 3±10 |
| 3 | Winter to spring | −25±13 | 38±1 | −63±13 |
|  | Spring to summer | 68±37 | −19±1 | 87±37 |
|  | Summer to autumn | −54±38 | 28±3 | −82±38 |
|  | Autumn to winter | 2±13 | −16±2 | 18±13 |
| 4 | Winter to spring | −18±13 | 31±2 | −39±13 |
|  | Spring to summer | 12±7 | −12±1 | 24±7 |
|  | Summer to autumn | −3±8 | 19±1 | −22±8 |
|  | Autumn to winter | 3±10 | −11±1 | 14±10 |

**Table S3 | Diagnostic results for the South China Sea basin, the Arabian Sea basin, and the Pearl River plume using the semi-analytical diagnostic framework for an OceMar regime. δDIC*_NO3_ and δDIC*_PO4_ indicate the net consumption of DIC relative to NO_3_ (Eq. (10)) and PO_4_ (Eq. (11)), respectively. Δ*p*CO2_NO3 (Eq. (13)) and  Δ*p*CO2_PO4 (Eq. (14)) indicate the sea-air Δ*p*CO_2_ estimated based on δDIC*_NO3_ and δDIC*_PO4_, respectively. ΔNO_3_/ΔPO_4_ indicates the apparent biological consumption of NO_3_ relative to PO_4_ obtained by Eq. (8)/Eq. (9) (see ‘Methods’ section). The uncertainties represent one standard deviation for each term largely due to the spatial variability of observations.**

| Case  study | Sampling  Time | Sampling  season | δDIC*_PO4_  (μmol kg^-1^) | δDIC*_NO3_  (μmol kg^-1^) | Δ*p*CO2_PO4  (μatm) | Δ*p*CO2_NO3  (μatm) | ΔNO_3_/ΔPO_4_ |
| --- | --- | --- | --- | --- | --- | --- | --- |
| South China Sea basin | Nov 9-22, 2010 | Autumn | 6.9±2.4 | 5.5±2.6 | 12.8±4.4 | 10.3±4.9 | 19.4±1.9 |
|  | May 28-Jul 14, 2014 | Summer | 16.9±9.4 | 19.9±14.6 | 31.6±17.7 | 34.5±28.4 | 14.2±1.9 |
| Arabian Sea basin | Jan 8-Feb 1, 1995 | Winter | 22.8±3.1 | 30.8±6.5 | 36.7±5.0 | 49.5±10.2 | 15.0±0.8 |
|  | Mar 14-Apr 8, 1995 | Spring | 3.7±4.6 | 10.5±6.8 | 5.7±7.2 | 16.2±10.5 | 14.5±2.3 |
|  | Jul 18-Aug 13, 1995 | Summer | 20.1±4.9 | 27.4±7.4 | 29.7±7.0 | 40.5±10.6 | 14.9±0.8 |
|  | Oct 29-Nov 25, 1995 | Autumn | 25.3±5.6 | 22.8±9.4 | 37.1±8.0 | 33.3±13.4 | 17.4±2.3 |
|  | Nov 30-Dec 26, 1995 | Winter | 25.7±7.8 | 30.6±9.9 | 38.7±11.3 | 46.1±14.3 | 15.5±0.5 |
| Pearl River plume | Jun 30-Jul 8, 2008 | Summer | 18.5±13.6 | −6.0±13.4 | 34.6±25.8 | −12.0±26.3 | 57.1±36.8 |
|  | Jul 20-31, 2016 | Summer | 15.9±11.7 | −34.0±19.8 | 29.0±21.3 | −62.3±36.5 | 175.9±179.5 |

**4 Supplementary References**

1. Pierrot D, Neill C and Sullivan K *et al*. Recommendations for autonomous underway *p*CO_2_ measuring systems and data-reduction routines. *Deep-Sea Res. II* 2009; **56**: 512-22.
2. Zhai W, Dai M and Cai W-J *et al*. The partial pressure of carbon dioxide and air-sea fluxes in the northern South China Sea in spring, summer and autumn. *Mar. Chem.* 2005; **96**: 87-97.
3. Zhai W and Dai M. On the seasonal variation of air-sea CO_2_ fluxes in the outer Changjiang (Yangtze River) Estuary, East China Sea. *Mar. Chem.* 2009; **117**: 2-10.
4. Millero FJ, Degler EA and Sullivan DWO *et al*. The carbon dioxide system in the Arabian Sea. *Deep-Sea Res. II* 1998; **45**: 2225-52.
5. Cao Z and Dai M. Shallow-depth CaCO_3_ dissolution: Evidence from excess calcium in the South China Sea and its export to the Pacific Ocean. *Global Biogeochem. Cycles* 2011; **25**: GB2019, doi:10.1029/2009GB003690.
6. Wang G, Xie S-P and Qu T *et al*. Deep South China Sea circulation. *Geophys. Res. Lett.* 2011; **38**: L05601, doi:10.1029/2010GL046626.
7. Tian J, Yang Q and Zhao W. Enhanced diapycnal mixing in the South China Sea. *J. Phys. Oceanogr.* 2009; **39**: 3191-203.
8. Cao Z, Dai M and Zheng N *et al*. Dynamics of the carbonate system in a large continental shelf system under the influence of both a river plume and coastal upwelling. *J. Geophys. Res.* 2011; **116**: G02010, doi:10.1029/2010JG001596.
9. Du C, Liu Z and Dai M *et al*. Impact of the Kuroshio intrusion on the nutrient inventory in the upper northern South China Sea: insights from an isopycnal mixing model. *Biogeosciences* 2013; **10**: 6419-32.
10. Zhang J-Z. Shipboard automated determination of trace concentrations of nitrite and nitrate in oligotrophic water by gas-segmented continuous flow analysis with a liquid wave guide capillary flow cell. *Deep-Sea Res.* 2000; **147**: 1157-71.
11. Ma J, Yuan D and Liang Y. Sequential injection analysis of nanomolar soluble reactive phosphorus in seawater with HLB solid phase extraction. *Mar. Chem.* 2008; **111**: 151-9.
12. Rixen T, Haake V and Ittekkot V *et al*. Coupling between SW monsoon-related surface and deep ocean processes as discerned from continuous particle flux measurements and correlated satellite data. *J. Geophys. Res.* 1996; **101**: 28569-82.
13. Brand TD and Griffiths C. Seasonality in the hydrography and biogeochemistry across the Pakistan margin of the NE Arabian Sea. *Deep-Sea Res. II* 2009; **35**: 283-95.
14. Hansell DA and Peltzer ET. Spatial and temporal variations of total organic carbon in the Arabian Sea. *Deep-Sea Res. II* 1998; **45**: 2171-93.
15. Valsala V and Murtugudde R. Mesoscale and intraseasonal air-sea CO_2_ exchanges in the western Arabian Sea during boreal summer. *Deep-Sea Res. I* 2015; **103**: 101-13.
16. Memet V, Bülent G and Aysel B *et al*. Spatial and temporal variations in surface water quality of the dam reservoirs in the Tigris River basin, Turkey. *Catena* 2012; **92**: 11-21.
17. Ali SM. Behavior of selected hydrochemical indicators of the Euphrates River, Iraq. *Arab. J. Geosci.* 2015; **8**: 2831-40.
18. Cai W-J, Guo X and Chen C-TA *et al*. A comparative overview of weathering intensity and HCO_3_ flux in the world’s major rivers with emphasis on the Changjiang, Huanghe, Zhujiang (Pearl) and Mississippi Rivers. *Cont. Shelf Res.* 2008; **28**: 1538-49.
19. Cao Z, Dai M and Evans W *et al*. Diagnosing CO_2_ fluxes in the upwelling system off the Oregon-California coast. *Biogeosciences* 2014; **11**: 6341-54.
20. Redfield AC, Ketchum BH and Richards FA. The influence of organisms on the composition of seawater. In: Hill MN (ed). *The Sea*. Wiley, New York, 1963; 26-77.
21. Gan J, Cheung A and Guo X *et al*. Intensified upwelling over a widened shelf in the northeastern South China Sea. *J. Geophys. Res.* 2009; **114**: C09019, doi:10.1029/2007JC004660.
22. Gan J, Li L and Wang D *et al*. Interaction of a river plume with coastal upwelling in the northeastern South China Sea. *Cont. Shelf Res.* 2009; **29**: 728-40.
23. Milliman JD and Farnsworth KL. River Discharge to the Coastal Ocean, A Global Synthesis. Cambridge University Press, 2011.
24. Oltman RE. Reconnaissance investigations of the discharge and water quality of the Amazon River. Vol. **552**, US Government Printing Office, 1968.
25. Geyer WR, Beardsley RC and Lentz SJ *et al*. Physical oceanography of the Amazon shelf. *Cont. Shelf Res.* 1996; **16**: 575-616.
26. Nittrouer CA and DeMaster DJ. The Amazon shelf setting: tropical, energetic, and influenced by a large river. *Cont. Shelf Res.* 1996; **16**: 553-73.
27. Lefèvre N, Moore G and Aiken J *et al*. Variability of *p*CO_2_ in the tropical Atlantic in 1995. *J. Geophys. Res. Oceans* 1998; **103**: 5623-34.
28. Ternon JF, Oudot C and Dessier A *et al*. A seasonal tropical sink for atmospheric CO_2_ in the Atlantic Ocean: the role of the Amazon River discharge. *Mar. Chem.* 2000; **68**: 183-201.
29. Körtzinger A. A significant CO_2_ sink in the tropical Atlantic Ocean associated with the Amazon River plume. *Geophys. Res. Lett.* 2003; **30**: 2287, doi:10.1029/2003GL018841.
30. Cooley SR, Coles VJ and Subramaniam A *et al*. Seasonal variations in the Amazon plume-related atmospheric carbon sink. *Global Biogeochem. Cycles* 2007; **21**: GB3014, doi:10.1029/2006GB002831.
31. Subramaniam A, Yager PL and Carpenter EJ *et al*. Amazon River enhances diazotrophy and carbon sequestration in the tropical North Atlantic Ocean. *Proc. Natl. Acad. Sci.* *USA* 2008; **105**: 10460-65.
32. Devol AH, Forsberg BR and Richey JE *et al*. Seasonal variation in chemical distributions in the Amazon (Solimões) River: A multiyear time series. *Global Biogeochem. Cycles* 1995; **9**: 307-28, doi:10.1029/95GB01145.
33. DeMaster DJ and Pope RH. Nutrient dynamics in Amazon shelf waters: results from AMASSEDS. *Cont. Shelf Res.* 1996; **16**: 263-89.
34. Lewis E and Wallace DWR. Program Developed for CO_2_ System Calculations. ORNL/CDIAC-105, Carbon Dioxide Information Analysis Center, Oak Ridge National Laboratory, U.S. Department of Energy, Oak Ridge, TN, 1998.
